# 3D printed model for triple negative inflammatory breast cancer

**DOI:** 10.1186/s41205-022-00158-4

**Published:** 2022-11-03

**Authors:** Yu-Hui Huang, Todd M. Tuttle, Noelle Hoven

**Affiliations:** 1 Department of Radiology, University of Minnesota, 420 Delaware Street SE, Minneapolis, MN 55455 USA; 2 Department of Surgery, University of Minnesota, 420 Delaware Street SE, Minneapolis, MN 55455 USA

**Keywords:** 3D printing, Desktop vat polymerization, Stereolithography, Triple negative inflammatory breast carcinoma

## Abstract

**Background:**

Access to imaging reports and review of the breast imaging directly with a patient with breast cancer helps improve the understanding of disease extent and severity. A 3D printed breast model can further enhance a patient’s understanding and communication with the healthcare team resulting in improved patient comprehension and patient input with reduced treatment decision conflict. Furthermore, 3D printed models can facilitate training of residents and fellows involved in the diagnosis and treatment management of breast cancer.

**Case presentation:**

We present a 3D printed breast tumor model segmented from positron electron tomography/computed tomography and fabricated via desktop vat polymerization as proof of concept for treatment planning for a patient diagnosed with triple negative inflammatory breast carcinoma.

**Conclusion:**

We illustrate benefits and indications for 3D printing in the management of breast cancer and specifically inflammatory breast cancer in this case. Fabrication and implementation of 3D printed models enhances patient’s understanding and communication with the healthcare team regarding their condition, treatment options and anticipated outcomes. It provides personalized treatment planning by examining patient-specific pathology and the anatomic spatial relationships. Furthermore, 3D printed models facilitate medical education for trainees across disciplines involved in the patient’s care.

## Background

Inflammatory breast cancer (IBC) is a rare and aggressive clinical presentation that necessitates a prompt diagnosis with a multidisciplinary treatment approach. Like other invasive breast cancers, diagnosis requires imaging and tissue confirmation [1]. Although improved access to imaging reports and a review of the breast imaging directly with the patient help to improve understanding of the extent of disease, incorporating 3D printed breast models may further enhance a patient’s understanding of their breast cancer diagnosis. Numerous studies now report that patient specific 3D printed anatomical models improve patient understanding of medical conditions and treatment options [2–6]. Utilization of 3D printing in medicine is increasing, and the potential positive clinical implications and interest continue to gain momentum. Numerous applications are demonstrated using 3D printing in breast cancer management including improved understanding of the disease for the patient and the healthcare team reducing treatment decision conflict especially between mastectomy and breast conserving surgery, aid in surgical planning, and intraoperative guidance to optimize the oncological and aesthetic outcomes [2, 7, 8]. Furthermore, 3D printed breast models can be used to facilitate medical education for multidisciplinary trainees from radiology to surgery, oncology, and pathology.

## Case presentation

**Fig. 1:**
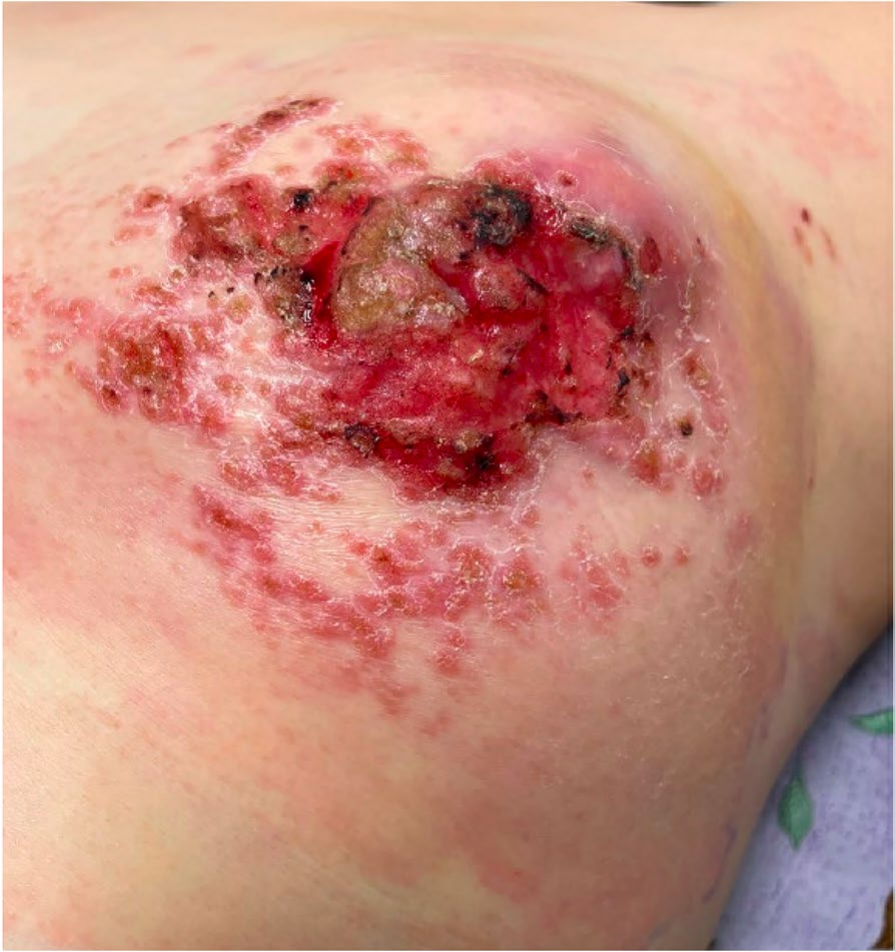
Clinical photograph of left breast after sustaining a human bite with erythema, swelling, and skin involvement

**Fig. 2:**
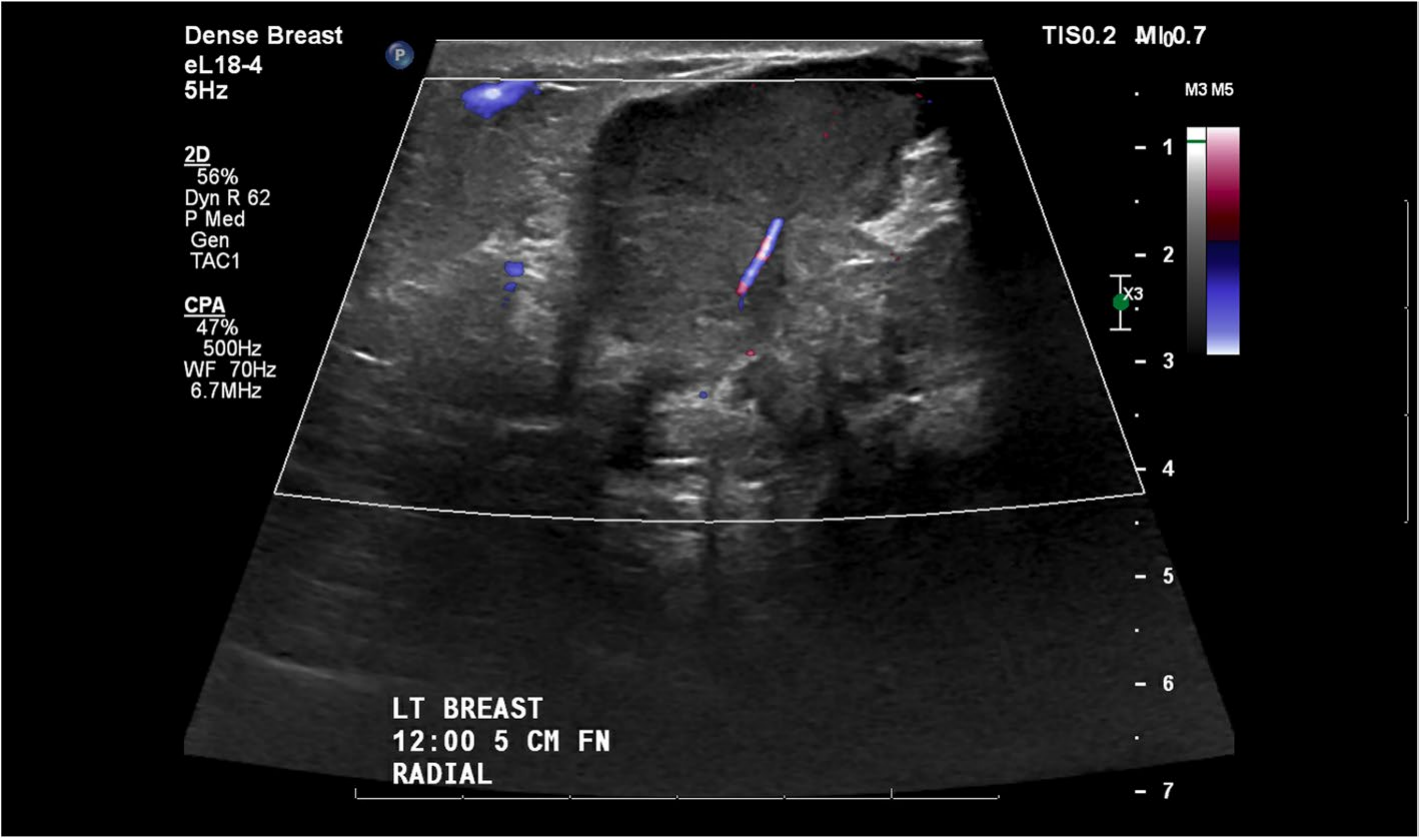
Targeted breast ultrasound demonstrates an irregular heterogenous hypoechoic mass with associated vascularity in left breast at the 12’o’clock position 5 cm from the nipple

**Fig. 3:**
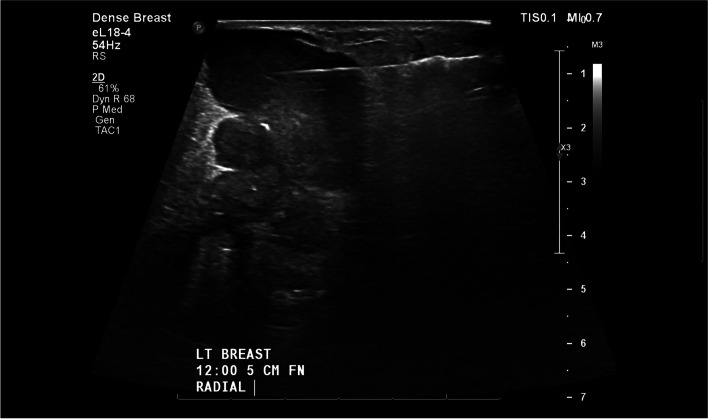
Ultrasound guided core needle biopsy of the irregular heterogenous hypoechoic mass with associated vascularity in left breast at the 12’o’clock position 5 cm from the nipple

**Fig. 4:**
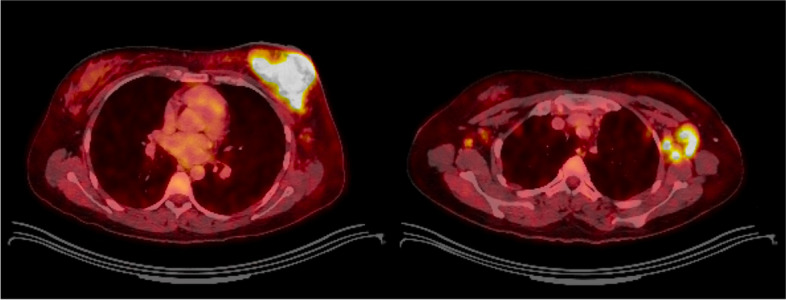
A staging positron emission tomography (PET)/computed tomography (CT) demonstrated a hypermetabolic 9.5 cm multilobulated centrally necrotic left breast mass with (**B**) extensive hypermetabolic left axillary and retropectoral lymphadenopathy. Nonenlarged hypermetabolic right axillary lymph nodes, were favored to be related to her recent documented COVID-19 vaccine administered in her right deltoid

A 29-year-old woman sustained a human bite on her left breast and presented with left breast pain, swelling and drainage after self-treatment with over-the-counter triple antibiotic ointment without improvement (Fig. 1). Her targeted breast ultrasound demonstrated an irregular heterogenous hypoechoic mass with associated vascularity in her left breast at the 12’o’clock position 5 cm from the nipple (Fig. 2). She underwent an ultrasound guided core needle breast biopsy (Fig. 3) which demonstrated carcinoma with squamous differentiation. Her subsequent left breast skin punch biopsy demonstrated invasive carcinoma involving the epidermis and dermis, supporting a diagnosis of invasive ductal carcinoma with focal features of metaplastic squamous cell carcinoma, Nottingham grade 3, which was estrogen, progesterone, and human epidermal growth factor receptor 2 (HER2) negative. Her staging positron emission tomography (PET)/computed tomography (CT) indicated a hypermetabolic 9.5 cm multilobulated centrally necrotic left breast mass with extensive hypermetabolic left axillary level 1 and 2 lymphadenopathy (Fig. 4). There was close approximation with the chest wall without evidence of chest wall invasion. There were also nonenlarged hypermetabolic right axillary lymph nodes, likely related to the recent documented covid vaccination administered in the patient’s right deltoid. The patient was ultimately diagnosed with stage 3C triple negative inflammatory breast carcinoma of her left breast.

**Fig. 5:**
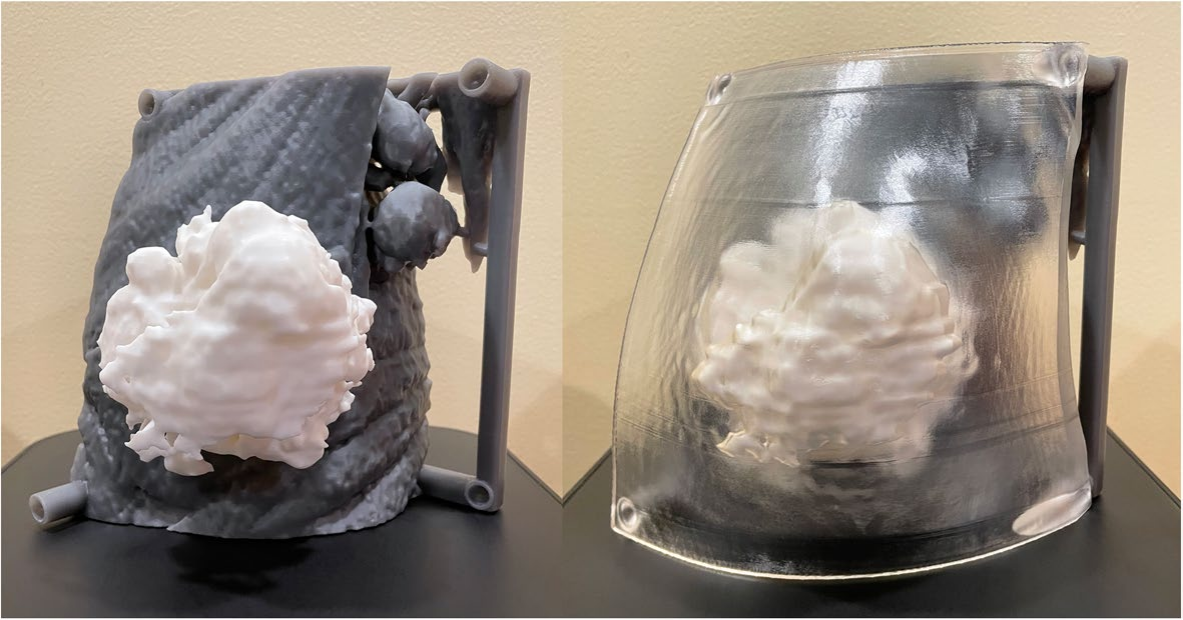
The breast model was printed with muscular chest wall and level 1 axillary lymph nodes in gray resin, tumor in white resin, and skin with ulceration in clear resin composited together with peg and hole system

The CT portion of the PET/CT (Biograph mCT64, Siemens Healthineers, Malvern, PA) was obtained with 1.0 mm slice thickness. The imaging file was downloaded as Digital Imaging and Communications (DICOM) files and imported into DICOM to PRINT® (D2P) (3D Systems, Inc. Rock Hill, SC) for segmentation to construct the 3D models. The skin, muscular chest wall and tumor took approximately 2 h to segment from the CT while referencing the PET data for the tumor. A peg and hole system was added to the model using Geomagic Freeform (3D Systems, Inc. Rock Hill, SC) to generate a complete model consisting of multiple independent components. The files were then exported as Standard Tessellation Language (STL) files, imported into proprietary 3D printing software (PreForm v3.21.0; Formlabs Inc. Somerville, MA), and printed using Formlabs Form 3B desktop 3D printer (Formlabs Inc. Somerville, MA). A life size breast model (Fig. 5) was printed with muscular chest wall including level 1 axillary lymph nodes (544 ml of gray resin), tumor (97 ml of white resin), and skin with ulcerations (154 ml of clear resin) with a total print time of 47 h and 25 min. Approximately 6 additional hours were allocated to postprocessing consisting of washes in isopropyl alcohol bath, post-curing, and support removal.

The 3D printed patient specific model demonstrates the spatial relationship between the breast tumor to the skin and underlying pectoralis muscles to highlight the tumor-to-breast ratio. The 3D printed model was provided during the consultation to assist with the treatment planning and decision-making process. The model assisted the patient in understanding the extent of disease. The patient completed 4 cycles of neoadjuvant pembrolizumab and chemotherapy of carboplatin and paclitaxel based on the KEYNOTE-522 trial followed by 4 cycles of pembrolizumab and Adriamycin with cyclophosphamide every 3 weeks [9]. After completion of neoadjuvant pembrolizumab and chemotherapy, follow-up PET/CT demonstrated findings compatible with a complete imaging response to treatment with resolved hypermetabolic left breast mass and lymphadenopathy. The left breast wound also improved with cessation of drainage. The patient underwent subsequent left simple mastectomy and left axillary lymph node biopsy. Surgical pathology results demonstrated no residual carcinoma after neoadjuvant pembrolizumab and chemotherapy with negative surgical margins. Three removed lymph nodes were negative for metastasis. The patient underwent adjuvant intensity-modulated radiotherapy of 50 Gy in 25 fractions to left chest wall with 10 Gy boost in 5 treatments to previously involved lymph nodes and mastectomy scar for locoregional control. The patient is doing well at the time of writing on adjuvant pembrolizumab.

## Discussion

The treatment planning and decision-making process for breast cancer is multifactorial and influenced by available information and resources for both the patient and the healthcare team. It has been established that imaging is vital to the diagnosis and treatment planning for patients with breast cancer, including IBC [1]. The patient specific 3D printed breast model derived from medical imaging can serve as an additional aid to enhance patient understanding regarding their own disease as well as the patient-physician communication regarding treatment options. In the presented case, the relevant breast anatomy was segmented from the CT portion of the staging PET/CT while referencing the information provided from the PET scan to ensure accurate tumor segmentation from normal fibroglandular tissue. To the authors’ knowledge, this technique has not been published for 3D printed breast tumor models. Studies by Santiago et al. and Galstyan et al. described techniques of 3D printed breast models segmented from contrast-enhanced breast magnetic resonance imaging (MRI) [2, 7, 8]. The interactive 3D printed model can help overcome potential educational, language and cultural barriers to improve one’s knowledge about their condition and enhance the quality of their decision making. Improved communication between the patient and the healthcare team has been shown to be associated with improved patient care and satisfaction [6, 10, 11]. Manipulation of ultrasound, mammograms, tomosynthesis (3D mammograms), and PET/CT is limited by the 2D display format and may not adequately display the spatial relationship between structures of interest. Patient specific 3D printed breast models segmented from patient’s volumetric data such as CT or MRI can enhance the visualization of the breast and tumor volume, the extent of the tumor and its spatial relationships to important anatomical structures such as the overlying skin, areola, and pectoralis musculature.

Barriers and limitations of 3D printed models include the upfront cost of acquiring a 3D printer, materials, segmentation software, the expertise required for the fabrication process and quality assurance, and the time needed for printing and post-processing. However, 3D printing has become more accessible with desktop vat polymerization technology making it more affordable with a smaller footprint printer while maintaining the high resolution needed for accurate fabrication of complex patient specific anatomy. The multicomponent breast model took approximately 53 h to complete, which occurred during pre-treatment planning and did not affect or delay patient care as the candidates selected for 3D printing are usually complex cases that require preoperative imaging acquisition for thorough diagnosis and treatment planning. With advancement of 3D printing technologies, production time and cost will continue to decrease.

## Conclusion

3D printing in breast cancer management is an emerging technology for personalized patient care. The patient specific 3D printed model can be utilized during consultation to provide a personalized visual and tactile representation of the patient’s breast and disease to facilitate the understanding of the condition and treatment options with the healthcare team. The 3D printed model may be used throughout the stages of care from the initial diagnosis and discussion of treatment options between the patient and the healthcare team to determine whether breast-conserving surgery is possible, to preoperative planning and pathologic correlation with the gross surgical specimen. An additional 3D printed model could also be fabricated to depict response to neoadjuvant chemotherapy which can then be reviewed with the patient. The model can also be utilized to improve the understanding and training of the resident and fellows across multiple disciplines who are involved in the patient’s care, particularly at an academic institution. Improved patient care and outcome may result from enhanced patient-physician communication and treatment planning through the use of patient specific 3D printed models. Further studies are needed to investigate how 3D printed models impact patient care and satisfaction, treatment outcomes, and pathologic correlation.

## Data Availability

The dataset used in the current study is available from the corresponding author on reasonable request.

## Abbreviations

IBC: Inflammatory breast cancer
2D: 2-Dimensional
3D: 3-Dimensional
HER2: Human epidermal growth factor receptor 2
PET: Positron emission tomography
CT: Computed tomography
DICOM: Digital Imaging and Communications
STL: Standard Tessellation Language
MRI: Magnetic Resonance Imaging
